# Development of Whole Slide Imaging on Smartphones and Evaluation With ThinPrep Cytology Test Samples: Follow-Up Study

**DOI:** 10.2196/mhealth.9518

**Published:** 2018-04-04

**Authors:** Yu-Ning Huang, Xing-Chun Peng, Shuoxin Ma, Hong Yu, Yu-Biao Jin, Jun Zheng, Guo-Hui Fu

**Affiliations:** ^1^ Department of Pathology Center Shanghai General Hospital Shanghai Jiao Tong University School of Medicine Shanghai China; ^2^ Faculty of Basic Medicine Shanghai Jiao Tong University School of Medicine Shanghai China; ^3^ TerryDr Info Technology Co, Ltd Nanjing China

**Keywords:** mobile health, image processing, cloud computing, whole slide imaging

## Abstract

**Background:**

The smartphone-based whole slide imaging (WSI) system represents a low-cost and effective alternative to automatic scanners for telepathology. In a previous study, the development of one such solution, named scalable whole slide imaging (sWSI), was presented and analyzed. A clinical evaluation of its iOS version with 100 frozen section samples verified the diagnosis-readiness of the produced virtual slides.

**Objective:**

The first aim of this study was to delve into the quantifying issues encountered in the development of an Android version. It should also provide insights into future high-resolution real-time feedback medical imaging apps on Android and invoke the awareness of smartphone manufacturers for collaboration. The second aim of this study was to further verify the clinical value of sWSI with cytology samples. This type is different from the frozen section samples in that they require finer detail on the cellular level.

**Methods:**

During sWSI development on Android, it was discovered that many models do not support uncompressed camera pixel data with sufficient resolution and full field of view. The proportion of models supporting the optimal format was estimated in a test on 200 mainstream Android models. Other factors, including slower processing speed and camera preview freezing, also led to inferior performance of sWSI on Android compared with the iOS version. The processing speed was mostly determined by the central processing unit frequency in theory, and the relationship was investigated in the 200-model simulation experiment with physical devices. The camera preview freezing was caused by the lag between triggering photo capture and resuming preview. In the clinical evaluation, 100 ThinPrep cytology test samples covering 6 diseases were scanned with sWSI and compared against the ground truth of optical microscopy.

**Results:**

Among the tested Android models, only 3.0% (6/200) provided an optimal data format, meeting all criteria of quality and efficiency. The image-processing speed demonstrated a positive relationship with the central processing unit frequency but to a smaller degree than expected and was highly model-dependent. The virtual slides produced by sWSI on Android and iOS of ThinPrep cytology test samples achieved similar high quality. Using optical microscopy as the ground truth, pathologists made a correct diagnosis on 87.5% (175/200) of the cases with sWSI virtual slides. Depending on the sWSI version and the pathologist in charge, the kappa value varied between .70 and .82. All participating pathologists considered the quality of the sWSI virtual slides in the experiment to be adequate for routine usage.

**Conclusions:**

Limited by hardware and operating system support, the performance of sWSI on mainstream Android smartphones did not fully match the iOS version. However, in practice, this difference was not significant, and both were adequate for digitizing most of the sample types for telepathology consultation.

## Introduction

With the data quality and speed improvements of automated microscopes and whole slide scanners [1,2], telepathology has become a major component in pathology labs [3,4]. Providing remote interpretation of digitized microscopic images and virtual whole slides, it allows diagnosis without physical transportation of samples but just data transfer over the internet. Telepathology not only greatly reduces the financial and time cost but also improves the availability and accessibility of priceless expert resources [5,6].

The reliability and practical value of virtual slides compared with the traditional glass version have been extensively assessed and recognized [7-9]. However, the high financial cost of whole slide imaging (WSI) solutions, and especially the up-front portion, has not seen a significant reduction after years of maturity, limiting its penetration into developing countries and regions or remote hospitals in the developed world [10-12]. As supplements for these situations, where limited manual operating is preferred over expensive automation, manual and low-cost alternatives of automated WSI have been studied and developed [13,14].

Ideally, utilizing tools that have been previously available to pathologists would reduce the cost of accessing WSI to its lowest and not require much operational training. Previously, we reported the development and clinical evaluation with frozen section samples of one such solution named scalable whole slide imaging (sWSI), a WSI system on smartphones [15]. With a mainstream smartphone mounted on the eyepiece of any optical microscope, a pathologist can scan the whole slide into a virtual copy by simply operating the microscope following this normal examination procedure. The image quality, based on the clinical evaluation results, is considered on par with high-end whole slide scanners for most tissue types, as assessed by senior pathologists, and its speed has been proven to be adequate for general applications.

However, the potential and assessment of smartphone-powered WSI has not yet been fully explored. On the one hand, the previous clinical evaluation was limited to working with the cryosection. Little evidence and discussion exists on the challenge of manually scanning frozen section samples compared with other types, although they have been considered among the most difficult for automatic scanners due to the unevenness and folding. On the other hand, during approximately 10 months of a public beta test in China, less than 10% of the approximately 2000 sWSI users selected the previously reported iOS version, reflecting Android’s dominance in developing markets and the practical value of sWSI on Android. This version, which possessed very different hardware and software configurations compared with the iOS one, is worth its own discussion and evaluation.

Cytology, the branch of pathology that studies and diagnoses diseases on the cellular level [16], introduces different types of challenges in the digitization revolution compared with its sister area of histopathology, which studies tissues and commonly employs cryosections for sample preparation [17-19]. Because cells are obviously smaller than the tissues that they compose, higher magnification power is required for their examination [20-23]. Consequently, the obstacles in scanning cytology samples through microscopes with smartphones include a stricter requirement of image quality, more frequent adjustment of the z-axis for focus, and varied image patterns. These characteristics make cytology samples a good test bench for a follow-up study of the clinical performance of sWSI.

In this paper, the development of sWSI on Android is reported, following up on the previous research and development of its simpler iOS version. The discussion and tests focused on camera data format optimization and factors limiting processing speed and user experience, particularly the effect of theoretical central processing unit (CPU) performance on processing speed. Emerging from model-specific hardware and firmware characteristics, and varying greatly among the hundreds of main-stream Android models, these issues can be common in developing medical imaging apps on Android with high image resolution, heavy computation, and real-time feedback. They are likely on an unavoidable path to the future of mobile health care, which is extending into image-based and artificial intelligence–powered apps. These results and discussion may provide future developers with precaution and guidance and draw the attention of hardware manufactures. On the clinical side, a follow-up clinical evaluation of sWSI scanning the ThinPrep Papanicolaou test samples was conducted and reported.

## Methods

### Review of the System Architecture, Core Algorithms, and Evaluation Results of Scalable Whole Slide Imaging on iOS

**Figure 1:**
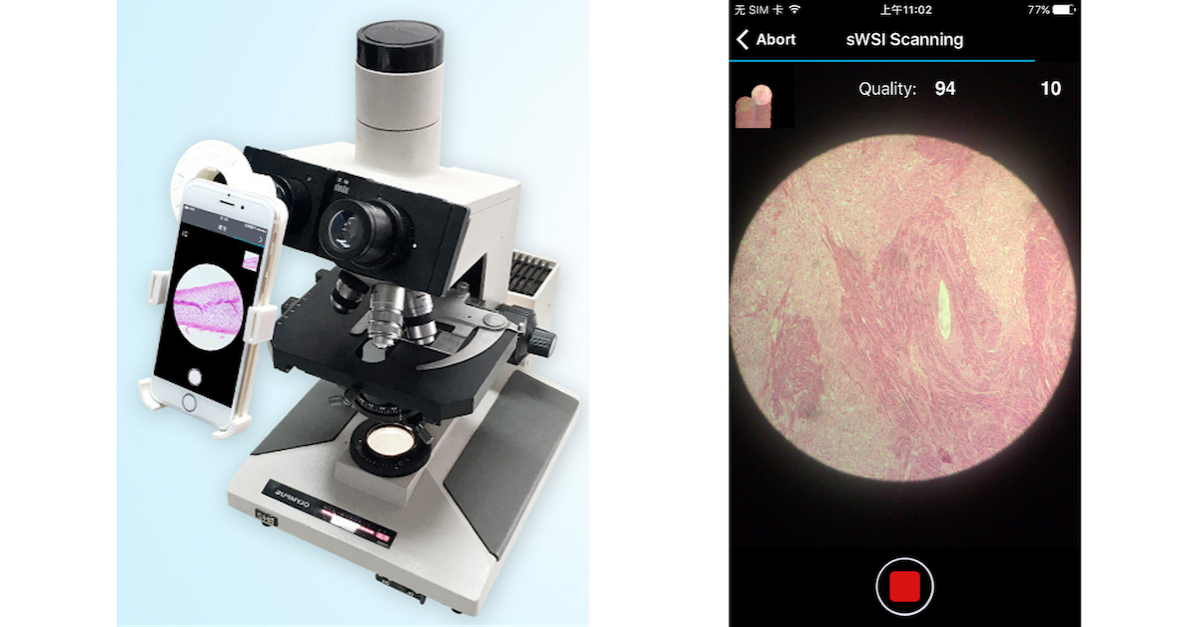
Typical hardware setup (left) and user interface (right).

**Figure 2:**
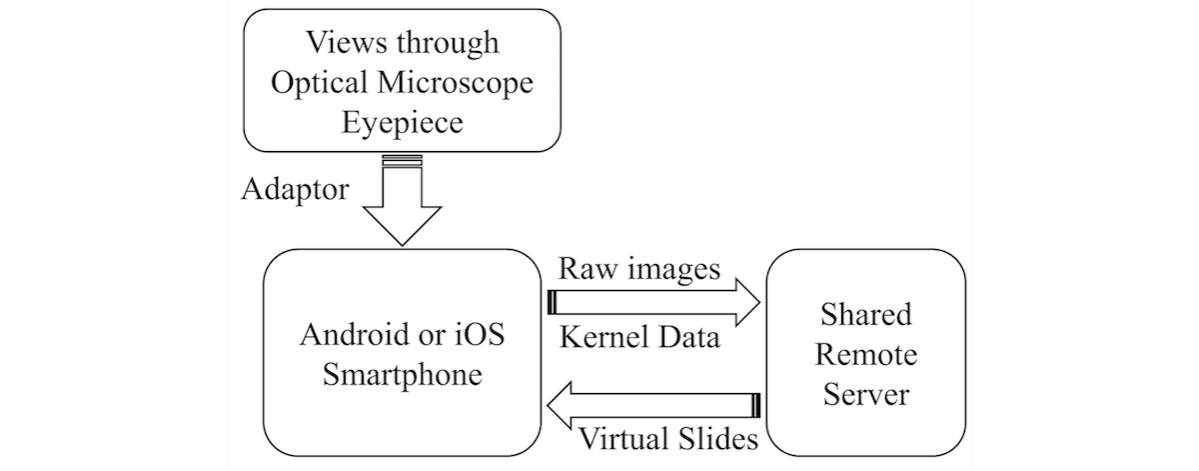
Simplified scalable whole slide imaging solution structure.

The sWSI solution is designed to provide affordable whole slide scanning service by leveraging existing optical microscopes with computer vision algorithms and universal availability of smartphones. The physical setup involves installing smartphones onto microscopes and aligning the cameras with the eyepieces, such as the one with a 3D-printed adaptor (demonstrated in Figure 1). During a scan, the user manually operates the microscope, whereas the sWSI app utilizes the image capturing functionality and just-enough computing power of smartphones to capture high-resolution images, process with approximation, and give smooth real-time feedback. Most of the computation burden is transferred to high-performance remote servers asynchronously, and the gigapixel virtual slides can be viewed with internet browsers, similar to digital maps. The simplified sWSI solution structure is illustrated in Figure 2.

There are 3 major algorithms that are designed to implement this 2-stage distributed computation model. First, an algorithm based on Speeded Up Robust Features key point detection and matching [24] tracks the location of the current field of view by stitching it with the last one to obtain and accumulate the relative movement. This is performed with down-sampled images to trade spatial accuracy for temporal efficiency. Second, the high-resolution field of views are transferred to cloud servers and restitched at full resolution for maximal accuracy. The stitching parameters of the down-sampled copies are then used to ensure restitching success. Finally, the highly nonlinear distortions introduced by smartphone camera lenses are corrected on-the-fly by solving a high-order polynomial model and projecting the images reversely. The model parameters are estimated from the matched key point pairs.

In the previous clinical evaluation, 100 frozen section slides were scanned with 20× objectives into virtual slides with sWSI on iOS, and examined by pathologists of the Pathology Center, Shanghai General Hospital/Faculty of Basic Medicine at the School of Medicine of Shanghai Jiao Tong University (SJTU-SMPC). The respective sample-wise diagnostic accuracies, using optical microscopy diagnosis by senior pathologists as the ground truth, were .78, .88, .68, and .50 for breast, uterine corpus, thyroid, and lung samples, respectively. The overall image quality is regarded by participating pathologists as generally on par with high-end scanners and not affecting diagnosis in most cases.

**Figure 3:**
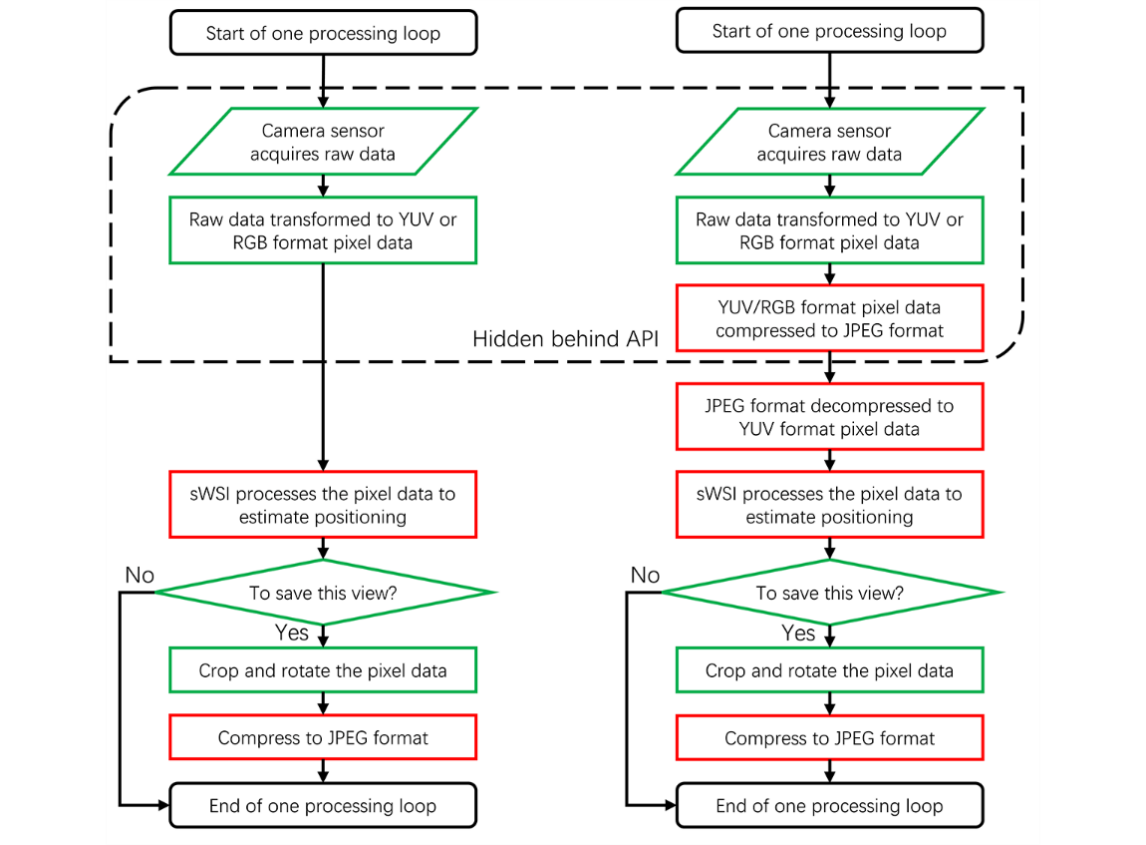
Image capturing and processing workflow on iOS (left) and Android (right). Computation-heavy steps are marked in red and lighter steps are marked in green.

### Optimizing Camera Data Format for High-Resolution Imaging on Android

In contrast to the proprietary iOS system on iPhones, Android is an open-source operating system that can not only be modified to a great extent but also operate on a wide variety of hardware beyond smartphones. On the positive side, this leads to a far greater number of smartphone models running Android differing in hardware specifications. Some models have retail prices of less than US $100, yet they are theoretically capable of running the same software apps on the flagship gadgets. This diversity greatly extends the user base of smartphones, specifically in developing countries, paving the way for the worldwide delivery of eHealth services.

On the negative side, the diversity posts significant design and engineering challenges, leading to higher development costs and occasionally limiting functionality. On the Android platform, the software apps need to adapt to the operating system environment and functionality at runtime. The large Android operating system family follows baseline specifications as defined by each version of Android software development kit, and they can have very different implementations and characteristics. In addition, manufacturers may keep the most efficient but private application programming interfaces to be only accessible to software that are bundled with operating systems to lock customers into their service ecosystem.

As a hardware- and firmware-dependent component, camera drivers in Android are implemented in native code and are only indirectly accessible dynamically via public application programming interfaces. As opposed to the iOS system, where all predefined data formats are usable, Android requires a determination of their availability at runtime. As of Android software development kit version 25, the only mandated implementation of data format for photo capturing is JPEG, a compressed and not directly processable format. Because sWSI requires real-time processing of each captured view to track the positioning and provides instant feedback to users, being forced to process such a compressed data format leads to additional computationally expensive compression-decompression steps in the workflow and dramatically increases overhead, as demonstrated in Figure 3.

One intuitive alternative is to confirm whether the operating system offers pixel data in YUV or red-green-blue (RGB) format, which can be directly processed, in order to select a shorter workflow similar to iOS. Unfortunately, among the 200 popular Android models tested during the development, few models support this approach, and the issue falls into one of the following 3 categories. In the first group, alternative formats are denied outright on some models. Among the second group, direct pixel data formats are provided but only with inadequate resolution [24]. Finally, where sufficiently high resolution is available, the images are often trimmed down horizontally to an aspect ratio of 16:9 compared with the standard 16:12 ratio, most likely intended for recording high-definition video only. For capturing photos, this would significantly restrict the field of view by up to 25% (see Multimedia Appendix 1)

Even among the remaining few that are usable, data structures often lack standardization. Specifically, the byte array of pixel data often has padding structures whose specification is not accessible in a standard application programming interface. For example, assume a small image with a resolution of 4 pixels in width by 4 pixels inches in height. Representing this image in YUV 4:2:0 format with an 8-bit quantization would yield the byte-array structure as demonstrated in Figure 2, where Y_ij_ is the Y component of pixel (i,j), U_ij_ and V_ij_ are respectively the U and V components shared by pixel (i,j), (i+1,j), (i,j+1), (i+1,j+1). There is one Y component byte for each pixel and a pair of U and V components for each set of 4 adjacent pixels; thus, there are 4×4, that is, 16 bytes for the Y-plane followed by 4×4/4, that is, 4 bytes for the UV-plane. However, on some phone models, several bytes are padded to the end of each row, column, or plane of pixels, mostly likely for a better efficiency of image compression, such as making the padded width and height multiples of 16 in JPEG. However, whether the byte array structure is padded into this multiple of 16 or not can be determined by calculation and validated by the fact that all padded bytes are 0s. This approach is guaranteed by any official documents. Considering that only 1 out of the 20 Android models used during development fits this category, the released sWSI app for Android supports the universal, but inefficient, JPEG format for image capturing.

### Considerations on Developing Medical Imaging Apps on Android With Heavy Computation

The diagnostic utility of real-time medical imaging apps such as sWSI is dependent primarily on image quality, which has been widely proven across apps in different medical branches and smartphone models [25-27]. From a practical perspective, user experience, and particularly user-perceived rate of data throughput is rarely emphasized. This is a trivial issue in static imaging or video recording, such as for teledermoscopy or ophthalmoscopy, but has emerged with great significance in sWSI and likely in future apps with heavy real-time computation.

On the basis of the feedback from clinical users of sWSI on Android, we found that there are 2 ways in which the user-perceived smoothness is impaired. On the one hand, there can be frequent freezing of the user interface or a constantly low refresh rate of the camera preview. On the other hand, some phone models suffer from a low update rate of the mini-map of scanned areas and an inability to keep up with faster movement of view. Apart from the image format-related driver issue discussed above, there are other factors that may have contributed to the great variance in operating smoothness, namely, the nondata characteristics of the camera driver and the processing unit.

Depending on hardware design and firmware implementation beyond the scope of this paper, camera drivers on Android smartphones significantly differ from each other in photo capturing overhead and lag. Here, we define the overhead as the time lapse between programmingly triggering the capture and receiving the image data. This adds delay to the whole processing loop, thereby reducing the throughput and update rate. The lag is defined as the lapse between the same trigger and when the camera preview unfreezes, as determined through a high-speed camera, with the overhead subtracted. It is out of the processing loop, but freezes the camera preview, causing no difference in speed but negatively affects the user experience. In extreme cases where processing time is as short as the lag, the preview would be permanently frozen.

The processing unit, particularly the main powerhouse of the CPU, intuitively has a strong effect on computation-hungry apps, such as sWSI. In practice, the case is very different from that for desktop computers due to the restriction on power supply and heat dissipation for mobile computing. Although the number of CPU cores ranges from 2 to 8 or more and the max CPU frequency from approximately 1.3 GHz to over 2.4 GHz, the sustainable performance for heavy computing varies far less dramatically. The increase in the number of cores is mostly intended to match computing power with dynamic workload by supporting high-frequency, power-draining cores with low-frequency, energy-saving ones. For the same reason, the boosted clock rate is similarly intended for short intervals only. Simultaneous activation of multiple cores at a high clock rate, although feasible, not only swiftly drains the battery life but also quickly leads to overheating, which forces the cores to slow down or even go offline in minutes. Ideally, spreading calculation into multiple cores at a lower frequency can be more efficient but requires system-level management, which is beyond the reach of third-party apps. As a result, sWSI on Android is optimized on single-threaded computation, and its evaluation includes the impact of max CPU single-core frequency on throughput.

### Technical Evaluation Setup

To verify the pervasiveness of the camera data format issue and CPU frequency’s impact on the processing speed of sWSI, tests were performed on 200 Android smartphones of popular models marketed after 2014. These physical devices were rented through Web-based testing platforms, such as TencentUTest (Dalian SJKP Technology Co. Ltd.), Testin.cn (Testin Information Technology Co. Ltd.), and Baidu MTC (Baidu Inc.), with 10% duplicates between platforms to check for consistency. A specific version of sWSI for Android with images for simulation is uploaded onto the devices, with logs recorded for measurement. Each test run starts with collecting available image formats and the size of the captured photo, if the raw pixel data format is supported. Next, it is kept running undisrupted for 10 min using the normal processing procedure, except that the captured photo data is replaced with looping simulation dataset. In the last minute, the average processing time of each view, excluding capture overhead, is recorded for comparison. This workflow is illustrated (see figures in Multimedia Appendix 1). The test process is repeated 3 times for each model, and the average is recorded.

### Clinical Evaluation Setup

**Figure 4:**
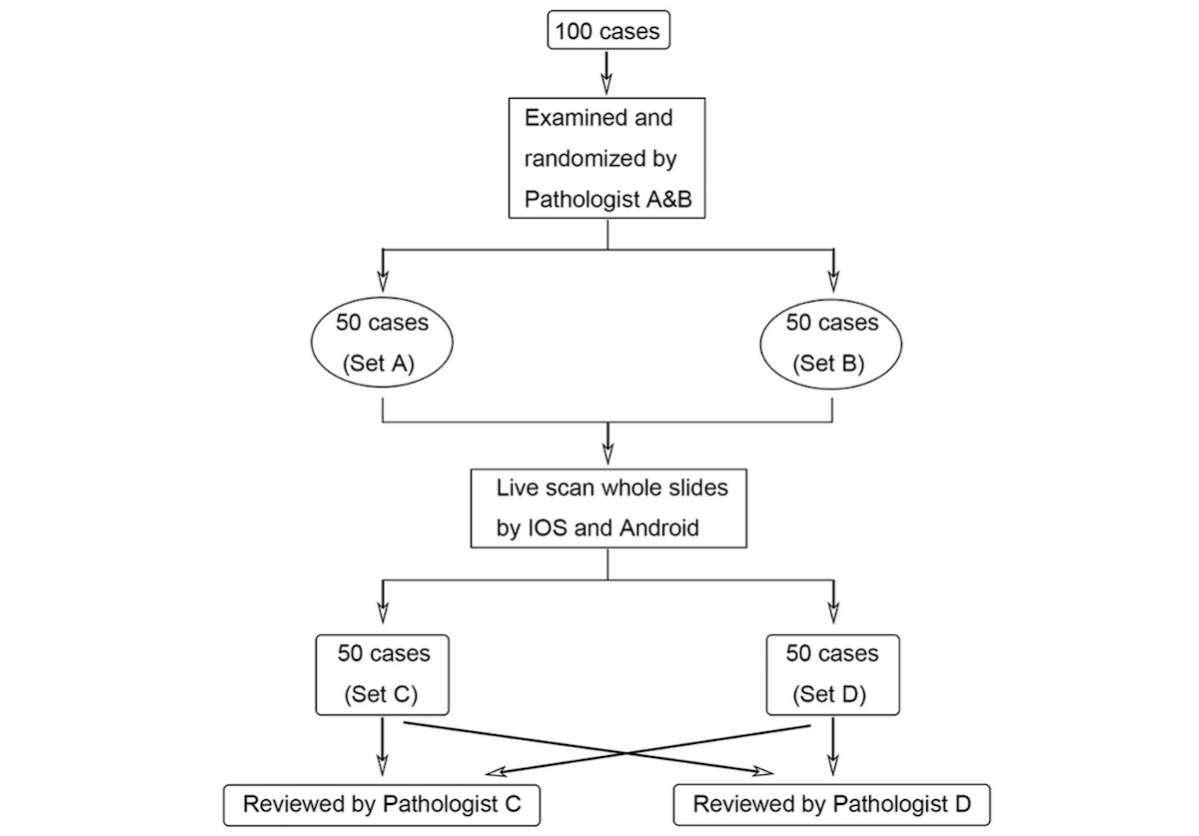
Clinical Evaluation Procedure of scalable whole slide imaging with ThinPrep cytology test samples.

To assess the diagnosis-readiness of virtual slides produced by both sWSI on iOS and Android in challenging cases, a clinical evaluation experiment was performed in the SJTU-SMPC from August 10 to September 3, 2017. A total of 100 TCT slides collected from SJTU-SMPC between January 1 and April 1, 2017 covering one of the 6 disease categories or the normal category, were randomly selected, as listed with proportions as shown in figures in Multimedia Appendix 1. These slides were prepared routinely by technicians in the department and may have issues such as unevenness and folding that are common for TCT. Examination with optical microcopy was used as the ground truth, and the sample set was split evenly into 2 groups and scanned with sWSI on Android and iOS, as summarized in Figure 4. The 2 iOS devices (iPhone 6 and iPhone 7) and the 2 Android handsets (HUAWEI mate8 and XIAOMI 5s) deployed in the experiment were purchased from the second-hand market with prices varying between US $120 and US $200. Three low-end bio-microscopes—an Olympus BH2-BHS, an Olympus CX21, and a Phoenix PH50-1B43L-PL—were employed for sWSI scanning, whereas a high-end Olympus BX51 was used for optical microscopy. Pathologists A, B, and D were all senior faculties from SJTU-SMPC, and pathologist C was a grade II trainee. The sWSI virtual slides were examined with a webpage-based pan-and-zoom tool on regular computer monitors without special color calibration.

The statistical metrics used in the study included accuracy, sensitivity, specificity, and Cohen's kappa coefficient (kappa). [28,29]. Accuracy was defined as the percentage of correctly classified patients. Sensitivity and specificity were defined as the proportion of people who truly have a designated disease or were truly free of a designated disease and were thus identified by the test, respectively. Cohen's kappa statistic quantifies the intermodality agreement into a single metric between 0.00 of no correlation and 1.00 of perfect match. In this study, the degree of agreement between diagnosis using conventional light microscopy and sWSI virtual slides was measured. All 4 measurements can be calculated using sample counts obtained by comparing the diagnosis to a gold standard, as with a 2 × 2 contingency table demonstrated in Table 1 and the formula in figures in Multimedia Appendix 1.

As a sideline study, 15 of the samples were scanned with Aperio AT2, a high-end scanner from Leica, to offer a direct comparison of image quality with sWSI. These virtual slides were not examined for accuracy.

**Table 1:** Assessment of diagnostic tests using 2 × 2 contingency table. sWSI: scalable whole slide imaging.

Gold standard	Positive (microscopes)	Negative (microscopes)	Total
Positive (sWSI)	True positive count: a	False positive count: b	a+b
Negative (sWSI)	False negative count: c	True negative count: d	c+d
Total	a+c	b+d	a+b+c+d

## Results

### Pixel Data Format Support on Android

The distribution of the tested Android model belonging to each camera data format issue category discussed is illustrated in figures in Multimedia Appendix 1. Statistically, only 3.0% (6/200) of the models met the standard of efficient high-resolution image capturing as supporting pixel data format.

- With resolution at least 1500 × 2000 pixels.
- Without trimming on the sides.
- Without padding or mismatch in the data sizes between the captured photos and that indicated by general definition.

Thus, 97.0% (194/200) of Android models would require approximately additional 100 ms time to process each high-resolution, full field of view photo captured due to unnecessary encoding or decoding caused by photo data format capability issue compared with iOS handsets. For cases requiring real-time reaction based on the processing feedback, this may introduce a significant lag as complained by many users of sWSI on Android.

### Max Central Processing Unit Frequency Versus Scalable Whole Slide Imaging Processing Time

**Figure 5:**
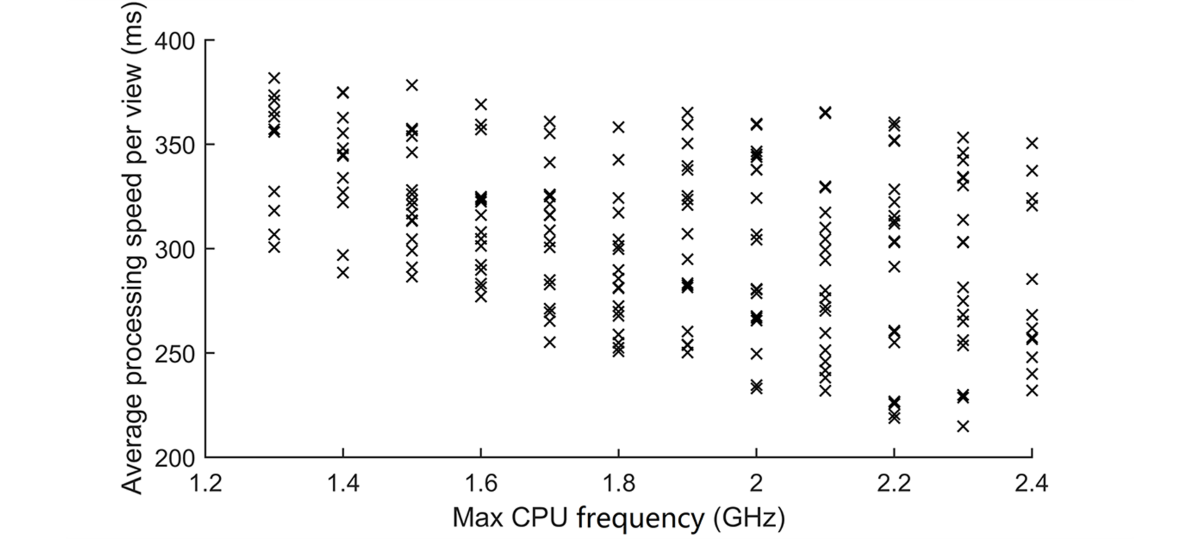
Max central processing unit (CPU) frequency versus average processing speed per view.

Figure 5 illustrates the average processing time per view on different phone models grouped into their theoretical max CPU frequency. As expected, a higher CPU frequency led to a faster processing speed, but the actual gain was more likely model-dependent, and cases where devices with a lower clock rate outperformed their theoretically faster counterpart were not rare. This is likely caused directly by the decrease in core frequency due to overheating after prolonged heavy computation, as observed in the limited number of models used in development. Fundamentally, the semiconductor device fabrication nodes determining heat production, the physical size of the device, and the industrial design of the internal structure may all contribute to this result, but they are purely matters of hardware, and deeper analysis is beyond the scope of this study.

### Diagnostic Concordance

Comparing telepathological diagnosis with the sWSI virtual slides and with optical microscopy, .70 and .82 kappa was respectively achieved by pathologists C and D. The scanning time per case averaged less than 20 min. The Acc, Sen, and Spe of each pathologist are illustrated in figures in Multimedia Appendix 1, and the overall results are summarized in Table 2. Importantly, significant variation in lesion recognition is commonly expected between different reviewers.

**Table 2:** Diagnosis concordance between those based on scalable whole slide imaging virtual slides and optical microscopy.

Disease	Observer							
	Pathologist C				Pathologist D			
	Accuracy	Sensitivity	Specificity	kappa^a^	Accuracy	Sensitivity	Specificity	kappa
High-grade squamous intraepithelial lesion	.83	.62	.80	.66	.91	.77	.91	.72
Low grade squamous intraepithelial lesion	.80	.54	.82	.56	.85	.69	.86	.70
Human papillomavirus	.89	.71	.92	.76	.95	.88	.94	.89
Atypical squamous cells of undetermined significance	.76	.63	.50	.51	.84	.75	.94	.67
Mycete	.82	.67	.67	.64	.96	.83	1.00	.90
Malignant melanoma	1.00	1.00	1.00	1.00	1.00	1.00	1.00	1.00
Normal	.82	.82	.66	.76	.94	.92	.90	.87
Average	.85	.71	.77	.70	.92	.83	.93	.82

^a^Cohen's kappa quantifies the intermodality agreement into a single metric between 0.00 (no correlation) and 1.00 (perfect match).

From a retrospective examination by the diagnosticians, the discrepancies in measurements should be primarily attributed to undercalled diagnoses with digital images. The most common mismatches noted in all data were between atypical squamous cells of undetermined significance or high-grade squamous intraepithelial lesions and low-grade squamous intraepithelial lesion, which may indicate poor reproducibility of the recognition of koilocytosis or koilocytoticatypia, followed by the discrepancy between low-grade squamous intraepithelial lesions and high-grade squamous intraepithelial lesions, suggesting a difficulty in the recognition of varying levels of cellular atypia. For example, in case 8, pathologist C and D examining sWSI virtual slides of atypical squamous cells were unable to exclude high-grade squamous intraepithelial lesion, whereas the glass-slide-based diagnosis was performed on a high-grade squamous intraepithelial lesion. Similarly, in case 2, the conventional glass-slide diagnosis was a high-grade squamous intraepithelial lesion, whereas one of the pathologists’ interpretation was atypical squamous cells from which high-grade squamous intraepithelial lesion cannot be excluded on either digital virtual slides, and another pathologist’s diagnosis with the Aperio system was low-grade squamous intraepithelial lesion.

The diagnosis statistics with Android and iOS smartphones are summarized in Table 3 (see figures in Multimedia Appendix 1). The respective accuracies are both 0.84 for pathologist C and between 0.89 and 0.92 for pathologist D. The average kappa is .7 for Android and .72 for iOS version of sWSI. These results indicated that the reliability of diagnosis made with virtual slides scanned by Android and iOS devices were both satisfying and not significantly distinguishable.

### Scalable Whole Slide Imaging Versus Aperio AT2

**Figure 6:**
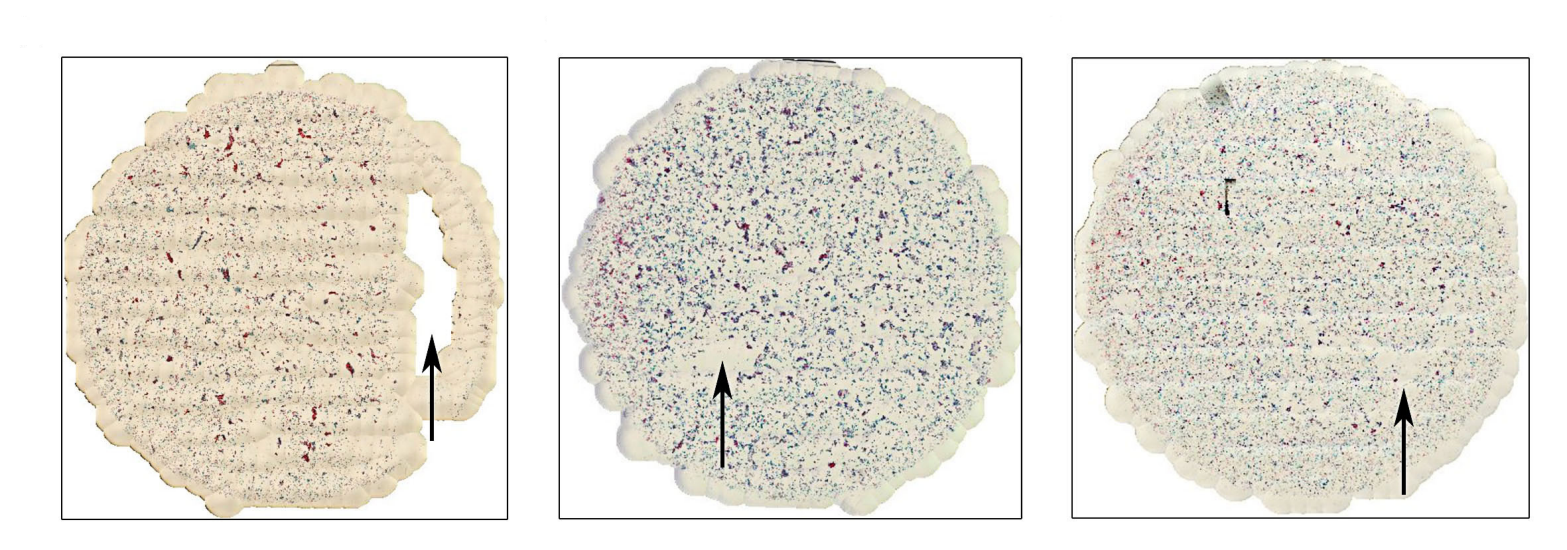
Cases no. 57 (left), no. 76 (center), and no. 98 (right).

**Figure 7:**
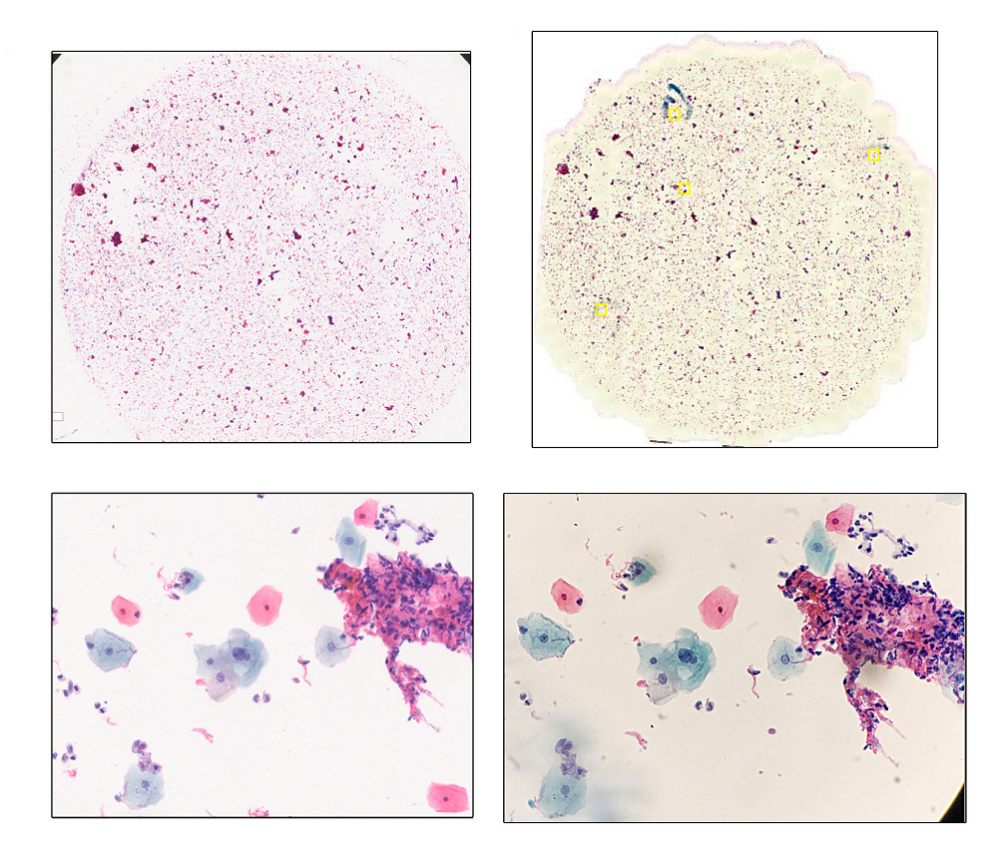
Case no. 35, the virtual slides (two on the top) and zoom-in regions (two on the bottom) from scalable whole slide imaging (sWSI) (two on the right) with good quality, compared with those from the Leica scanner (two on the left).

**Figure 8:**
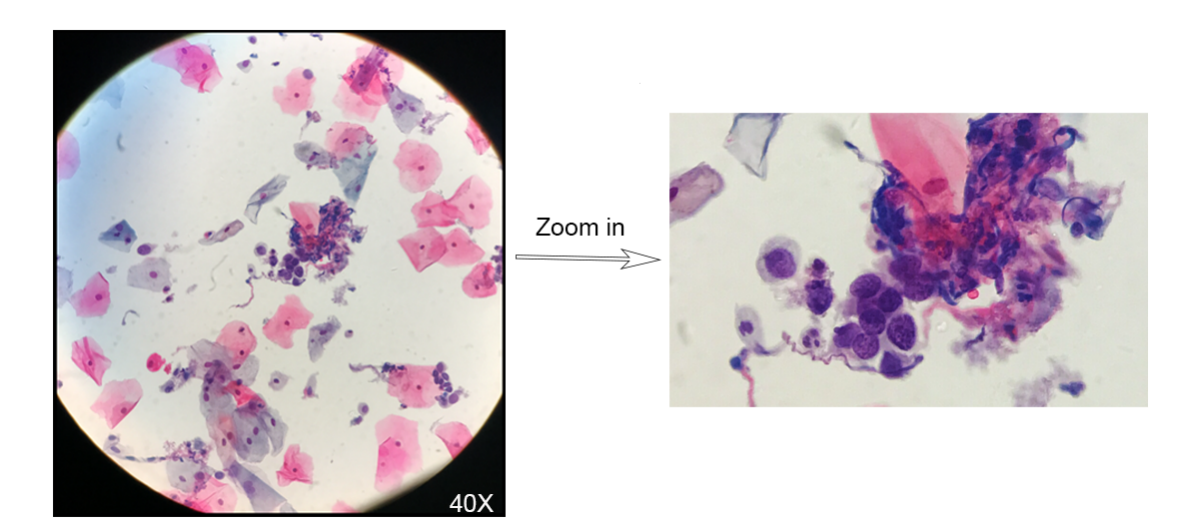
Typical views captured by a high-end whole slide scanner (right) versus a static view captured by scalable whole slide imaging (left), 40× magnification.

Among the 15 cases scanned by both sWSI and Aperio AT2, 2 were recognized as improperly prepared, with one blurred and the other incomplete. With the scanner, case #38 was deemed not scannable, and case #66 was seriously blurred. By checking the glass slide, it was found that 2 coverslips were placed on top of case #66, and likely confused the scanner’s autofocus. The same problem was avoided by manual sWSI scan (see figures in Multimedia Appendix 1). Although scanning with sWSI at lower magnification yields a lower resolution (Figure 6), compared with the scanner, the high magnification static field of views produced subjectively similar or better quality. Figures 7 and 8 show one such comparison with 40× magnification.

**Table 3:** Diagnosis concordance between scalable whole slide imaging (sWSI) based on Android or iOS and optical microscopy.

Observer	Android				iOS			
	Accuracy	Sensitivity	Specificity	kappa^a^	Accuracy	Sensitivity	Specificity	kappa
Pathologist C	.84	.67	.76	.55	.84	.71	.74	.66
Pathologist D	.92	.86	.89	.84	.89	.78	.84	.78
Average	.88	.77	.83	.70	.87	.75	.79	.72

^a^Cohen's kappa quantifies the intermodality agreement into a single metric between 0.00 (no correlation) and 1.00 (perfect match).

## Discussion

### Current Limitations

Although the sWSI solution is clinically recognized by pathologists and achieves its goal of trading full automation for saving financial cost by multiple orders, it suffers from a few technical weaknesses. Many weaknesses are caused by the inherent data model of the image stitching and distortion correction algorithm and thus may not be resolved with further development without switching to a different kernel. Others may be addressed in studies in the near future.

First, an underqualified sample preparation may limit the sWSI’s spatial coverage of samples. Specifically, uneven cell distributions and densities might cause TCT slides to be partially unscannable, such as the blank region of case #57 to the right of the image as shown in Figure 6. This may be caused by having too few cells located nearby, whereas a typical distribution would approximate those in Figure 6. In these cases, the image stitching algorithm determines that few reliable key points can be used for tracking, thus denying the views. This may lead to a loss of information in these cells and, consequently, inaccurate data analysis or diagnosis.

Second, the diagnosis error introduced by reviewer bias may have underrated the quality of the sWSI virtual slides. On the one hand, both reviewers of sWSI virtual slides rarely examined digital virtual slides in routine work and complained about the different perspectives between optical and virtual microscopy. On the other hand, it is widely known that the thresholds of judging ambiguous cases vary among pathologists. In retrospect, pathologists participating in the experiment indicated multiple cases with such ambiguity. However, standardization of diagnosis criteria can be difficult to establish, as decisions are currently rarely based on quantitative measurements.

Finally, although a majority of sWSI virtual slides show no significant difference in comparison with those produced by a high-end scanner (Figure 7), there are a few in which the sWSI virtual slides contained obvious misalignment of separate views and uneven brightness (Figure 6,) that are likely caused by an accumulation of tracking error and uncalibrated evenness of light source. These should be fixable with an improved distortion model.

### Future Work

On the basis of the reviews by senior pathologists from SJTU-SMPC of the results of a previous study and this study, sWSI has been clinically proven to be a legitimate alternative to automatic whole slide scanners. Its cost-effectiveness makes it a solid intermediate between localized optical microscopy and fully automatic but expensive scanners. However, there is room for improvement on the sWSI solution.

First, processing speed may be further enhanced. Putting hardware issues aside, there should be a number of methods to improve the image processing speed of sWSI, such as multithread optimization on more energy-efficient models and recrewing the general purpose graphics processing unit. The former technique is widely used on desktop computer programs but may not be practical on mobile devices for a sustained boost due to the power constraints discussed in the previous sections. However, it may be worth further investigation on newer models, whose energy efficiency has been increasing steadily due to advanced semiconductor technology. The latter has been tested on iOS with substantial gain but shown to be unstable on some versions of the operating system, as previously reported. Considering the advantage of the general purpose graphics processing unit in energy-efficient float-point computing, further study and development of its utilization on Android may also help with reducing overheating, thus yielding considerable gain in data throughput.

Second, the low-magnification-scan-and-high-magnification- static-view method can be further explored for productivity. In this version, the microscope operator is responsible for deciding where the static views are located and thus must have diagnostic knowledge at least on the level of a junior pathologist. This requirement may be lifted if a viewer of the virtual slides, most likely the senior professional from whom advice is sought, may interact with the operator, and mark the region instead.

Third, further testing is needed. Unlike the iOS version, sWSI on Android experiences a relatively high crash rate on specific devices after scanning approximately 500 views without detectable memory leakage or overflow. If this is related to factors other than the app’s functionality, then it may indicate some other engineering obstacles for other high-performance imaging apps on Android in general.

Finally, low-cost automation of microscopes may significantly improve productivity of the sWSI solution at an affordable cost. Although automated microscope stages based on step motors are mature and widely available, high positioning accuracy leads to high prices even for recent low-cost solutions [30,31]. Since sWSI tracks the field of view through software and computation instead of physical measurement, such constraints on accuracy may be greatly relaxed from the micron level to 100-micron level, thereby reducing the cost dramatically.

### Conclusions

In this paper, the follow-up development on the Android platform and clinical evaluation of sWSI, a WSI solution on a smartphone, is reported. Due to the diversity of handsets and operating system characteristics, several factors impair the theoretical performance and user experience of the Android version compared with the previously reported iOS model. However, in a clinical evaluation of challenging TCT samples, an insignificant difference was discovered between the diagnosis accuracy based on the virtual slides produced by either version. sWSI on both mobile platforms is recognized as a reliable tool for telepathology consultation and a competitive alternative to WSI scanners.

A major problem causing a slower processing speed of sWSI on Android is the rare support of high-resolution and reliable pixel data format of cameras. In tests on mainstream Android models, only 3.0% (6/200) provided pixel data format that can be used directly for processing with at least 3-megapixel resolution, full field of view, and no padding. On other handsets, the JPEG format, which is compressed and must be decompressed for processing, is the only reliable option. This encoding-decoding process is unnecessary and computationally expensive.

In addition, it was verified that although theoretical CPU performance as measured by max frequency varied greatly, the sustainable processing speed of the computation-heavy sWSI kernel was largely model-dependent. Although more sophisticated testing is required to determine the cause, an intuitive answer is CPU thermal throttle leading to a decrease in frequency. Another observed factor leading to user-experience degradation is screen freezing caused by a lag of resuming camera preview after capturing a photo, but its quantitative effect also requires further investigation beyond the plan of this study.

In the clinical evaluation conducted in SJTU-SMPC, a diagnosis based on sWSI virtual slides reached 87.5% (175/200) accuracy and a kappa value of .76 on average, with gold-standard optical microscopy used as the ground truth. The selected slides are TCT samples covering 6 diseases as well as normal samples that are intended to complement the frozen section samples used in the previous study, as they require finer details and are difficult to scan manually due to the varying intercell distance. After retrospectively examining the data, all senior pathologists from SJTU-SMPC considered sWSI’s performance on par with high-end scanners and highly suitable for smaller or remote hospitals with less frequent need for teleconsulting.

## Appendix

Multimedia Appendix 1 Supplementary figures.

## Abbreviations

CPU: central processing unit
SJTU-SMPC: Pathology Center, Shanghai General Hospital/Faculty of Basic Medicine at the School of Medicine of Shanghai Jiao Tong University
sWSI: scalable whole slide imaging
TCT: ThinPrep cytology test
WSI: whole slide imaging
