# Supplementary material for: Development of Whole Slide Imaging on Smartphones and Evaluation With ThinPrep Cytology Test Samples: Follow-Up Study
Source: JMIR Mhealth Uhealth. 2018 Apr 4;6(4):e82. doi: 10.2196/mhealth.9518 (PMC5906711; doi:10.2196/mhealth.9518)
Supplement: Multimedia Appendix 1 [file mhealth_v6i4e82_app1.pdf]

# Multimedia Appendix 1

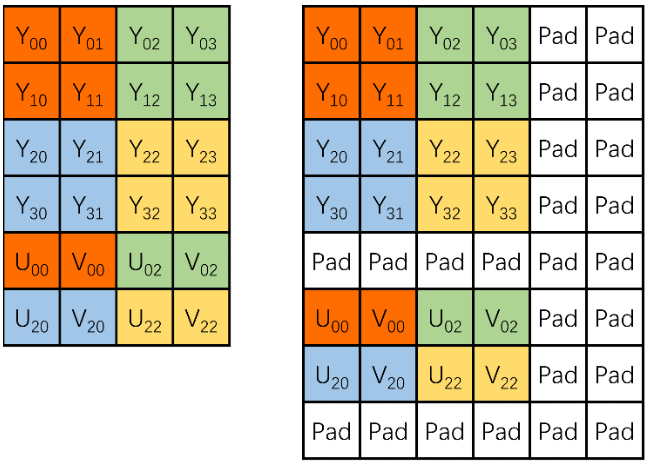


*Figure 9. Example of a 4-by-4 pixel image in YUV420 format with (left) and without (right) padding.*

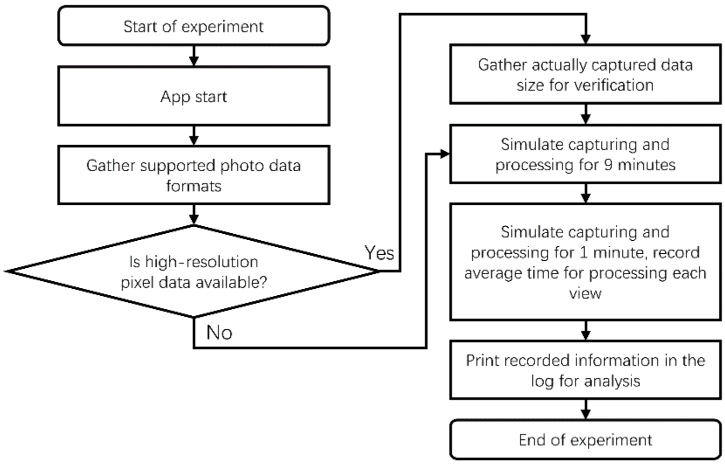


*Figure 10. Technical evaluation experiment workflow.*

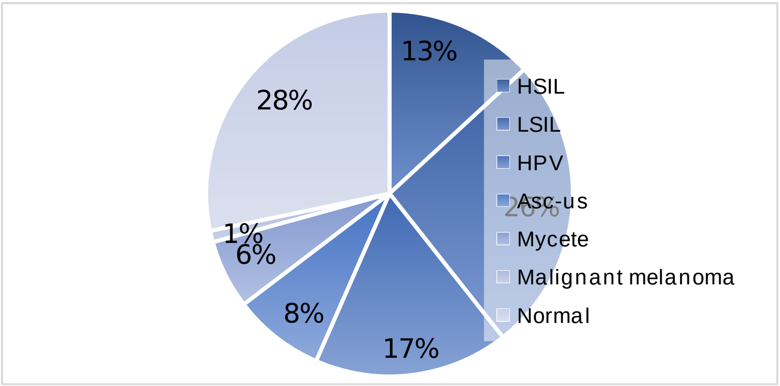

*Figure 11. Sample categories, counts and notes.*

$$\text{Accuracy} = \frac{a+d}{a+b+c+d}$$

$$\text{Sensitivity} = \frac{a}{a+c}$$

$$\text{Specificity} = \frac{d}{b+d}$$

$$\text{Kappa} = \frac{\text{Accuracy}-p_e}{1-p_e}, \text{ where } p_e = \frac{(a+b)(a+c)(c+d)(b+d)}{(a+b+c+d)^2}$$

*Figure 12. Statistical metrics formula using sample counts in Error: Reference source not found.*

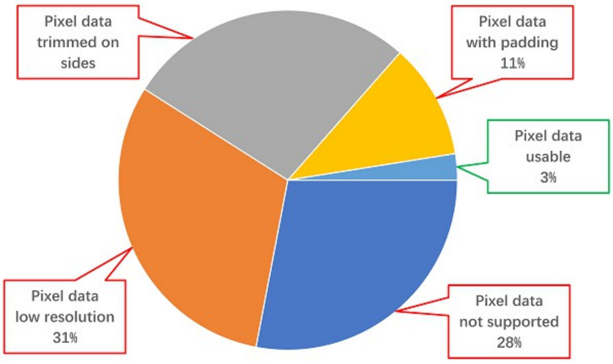


*Figure 13. Distribution of Android models with camera data format issue. Enclosed in red/green: not/being usable for high-resolution imaging.*

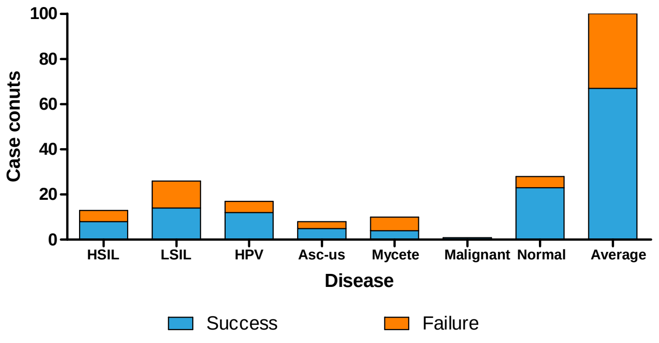


*Figure 14. Diagnosis accuracy of pathologist C based on sWSI VS.*

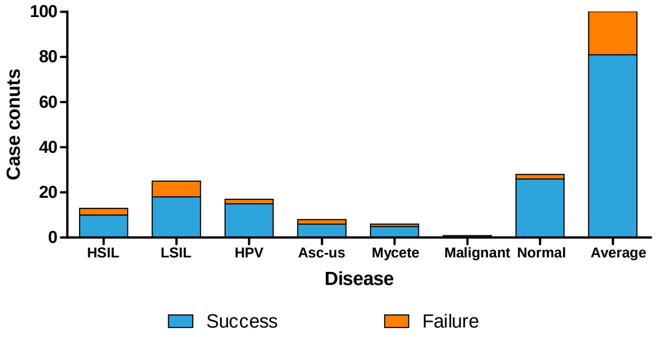


*Figure 15. Diagnosis accuracy of pathologist D based on sWSI VS.*

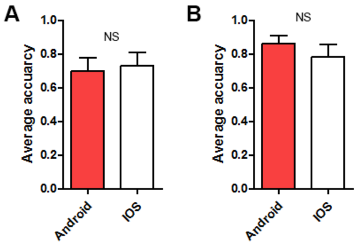


*Figure 16. Consistency between diagnosis made from VS scanned by sWSI on Android and iOS.*

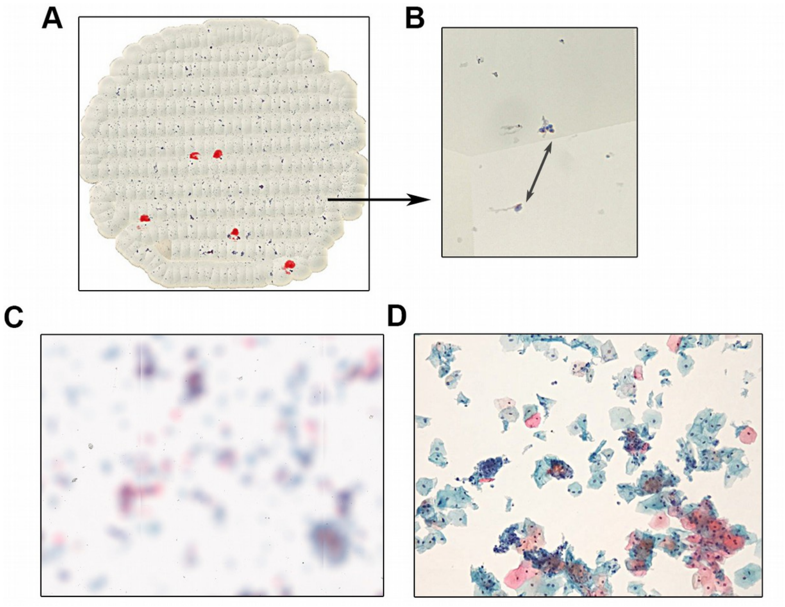


*Figure 1. Cases No. 38 and No. 66*

*(A) and (B) from PHONE, A represents the whole image, B displays a mosaic effect, the arrow indicates the distance between cells.*

*(C) from Aperio AT2, D represents the same slides as C, which is from PHONE.*
